# Distinct Thresholds and Interactive Effects of Environmental Drivers on Forest Gross Primary Productivity Across Temperate and Cold Climate Zones

**DOI:** 10.3390/plants15213428

**Published:** 2026-11-04

**Authors:** Weihua Liu, Yalin Hu, Shengzhao Xu, Xiaojie Li, Lili Feng, Rong Ge, Mengyu Zhang

**Affiliations:** 1 College of Forestry, Fujian Agriculture and Forestry University, Fuzhou 350002, China; liuwh@fafu.edu.cn (W.L.);; 2 Research Institute of Forestry, Chinese Academy of Forestry, Beijing 100091, China; 3 Institute of Natural Resources and Environmental Audits, School of Government Audit, Nanjing Audit University, Nanjing 211815, China; 4 Key Laboratory of Ecosystem Network Observation and Modeling, Institute of Geographic Sciences and Natural Resources Research, Chinese Academy of Sciences, Beijing 100101, China

**Keywords:** gross primary productivity, threshold effect, interactive effect, environmental change, explainable machine learning, SHapley Additive exPlanations

## Abstract

Forest gross primary productivity (GPP) is sensitive to environmental change, and assessing its complex responses is essential for evaluating ecosystem carbon sinks, yet the thresholds and interactions among driving factors remain unclear. By integrating long-term eddy covariance measurements with an optimal ensemble learning model and the SHapley Additive exPlanations algorithm, we identified the key environmental factors, threshold characteristics, and interactions driving daily forest GPP across the temperate climate zone (TCZ) and the cold climate zone (CCZ). Forest GPP exhibits clear nonlinear responses that differ across climate zones: solar radiation and air temperature are the dominant drivers of daily GPP variation in the TCZ and CCZ, respectively. In the TCZ, the threshold at which radiation switches from limiting to promoting GPP is estimated at 151.0 W m^−2^ (95% confidence interval: 149.6–152.5 W m^−2^), with thresholds of 12.3 °C (12.2–12.4 °C) for air temperature and 7.5 °C (7.3–7.6 °C) for soil temperature. In the CCZ, the corresponding thresholds are 156.0 W m^−2^ (153.7–158.1 W m^−2^), 9.1 °C (9.0–9.2 °C), and 8.6 °C (8.5–8.8 °C). Dominant factors operate primarily through main effects, whereas non-dominant factors exert stronger interaction effects. The direction of the radiation effect on GPP is modulated by temperature, and the radiation–temperature interaction is stronger in the cold than in the temperate climate zone. These findings clarify the nonlinear response characteristics and interaction regulation of forest GPP across climate zones, providing applicable insights for climate-adaptive forest management and carbon policy design.

## 1. Introduction

Vegetation gross primary productivity (GPP) represents the largest carbon flux component in the terrestrial carbon cycle and plays a crucial role in mitigating the rise of atmospheric carbon dioxide (CO_2_) concentration and curbing global warming. As the primary contributor to terrestrial carbon sequestration, forests have received special attention because their productivity is affected by increasing climate variability [1], which relates to the stability and sustainability of forest carbon sequestration functions [2,3]. Consequently, accurately estimating forest GPP and analyzing its driving mechanisms are essential for addressing climate change and formulating scientifically grounded carbon neutrality strategies.

Forest GPP is regulated by multiple environmental factors, including air temperature (TA) [4], precipitation (PRE) [5], solar radiation (RAD) [6], atmospheric drought [7], soil temperature (TS) [8], and soil water content (SWC) [9]. However, due to the significant spatial heterogeneity of environmental conditions, the relative importance of these factors differs significantly across climate zones. Research based on observational data has identified temperature as the primary driver of daily GPP variation in global forests [10]. In contrast, studies of subtropical forests suggest that radiation plays a more significant role than temperature [11,12]. Additionally, some investigations suggest that vapor pressure deficit (VPD) may be even more critical in governing forest GPP variation [13]. Despite these advances, existing studies suffer from two major limitations. First, the quantification of interaction effects among environmental factors remains insufficient. Although individual factors can directly constrain photosynthesis, they also influence GPP indirectly through complex interactions [14]. A recent study indicates that warming does not affect ecosystem functioning in isolation, but rather in concert with other global change drivers [15]. Few studies have quantitatively disentangled the main effects from interactive effects, leaving the mechanisms less well-known [13]. Second, the nonlinear responses and threshold effects of GPP to environmental drivers are often overlooked. When environmental changes exceed critical thresholds, their impacts on ecosystem function can change significantly [16]. Conventional approaches such as correlation analyses are inadequate to capture nonlinearity, thresholds, or driver interactions, introducing considerable uncertainty [13]. Therefore, there is an urgent need to systematically identify the dominant drivers, threshold characteristics, and interactive controls of forest GPP across contrasting climate zones.

As ecological observation data becomes increasingly abundant and machine learning (ML) models continue to advance, an increasing number of ecological studies are employing ML techniques to explore the multidimensional dependencies between environmental factors and the carbon cycle [10,12,13,17]. However, owing to the absence of an explicit functional mapping between inputs and outputs, ML methods often exhibit lower transparency than ecosystem process-based models [18]. To this end, several interpretability methods have been developed to uncover how driving variables influence model outputs, among which SHapley Additive exPlanations (SHAP) are widely used. Proposed by [19], SHAP provides a unified approach to interpreting ML models and has become increasingly popular in ecological studies for quantifying nonlinear relationships between environmental factors and carbon fluxes [20,21]. For example, [20] applied SHAP to explore the nonlinear effects of climate variables on carbon sinks in karst ecosystems, while [22] used SHAP to disentangle the relative influence of soil water supply and atmospheric water demand on terrestrial GPP. However, most existing SHAP-based studies have focused either on nonlinear response shapes or on ranking variable importance, with limited attention to quantitatively distinguishing main effects from interactive effects or to identifying specific threshold values of environmental drivers, especially across different climate zones. These gaps hinder a mechanistic understanding of how multiple factors jointly regulate forest GPP under contrasting environmental contexts.

The temperate climate zone (TCZ) and the cold climate zone (CCZ), defined following the Köppen–Geiger classification, cover approximately 13.4% and 24.6% of the global land surface, respectively, and they exhibit markedly divergent carbon sink trajectories and differential responses to ongoing climate change [1,23]. Although previous studies have advanced the understanding of dominant drivers of GPP in these zones [6,10,21], main effects of environmental drivers are rarely separated from their interactions across climate zones, and the thresholds at which these drivers shift from limiting to promoting GPP remain unclear. This study therefore focuses on forest GPP in two climatically contrasting zones: the TCZ and CCZ. By integrating a state-of-the-art ML model with the SHAP interpretability framework, we investigate the relationship between daily forest GPP and environmental drivers. Our objectives are to: (1) identify the dominant environmental factors influencing forest GPP in each climate zone; (2) quantify the response thresholds of GPP to key drivers; and (3) assess the interactive contributions among environmental drivers. Understanding how environmental drivers shift from limiting forest productivity to promoting it is therefore essential for designing adaptive carbon sequestration strategies.

## 2. Results

### 2.1. Validation of Gross Primary Productivity Simulation

**Figure 1:**
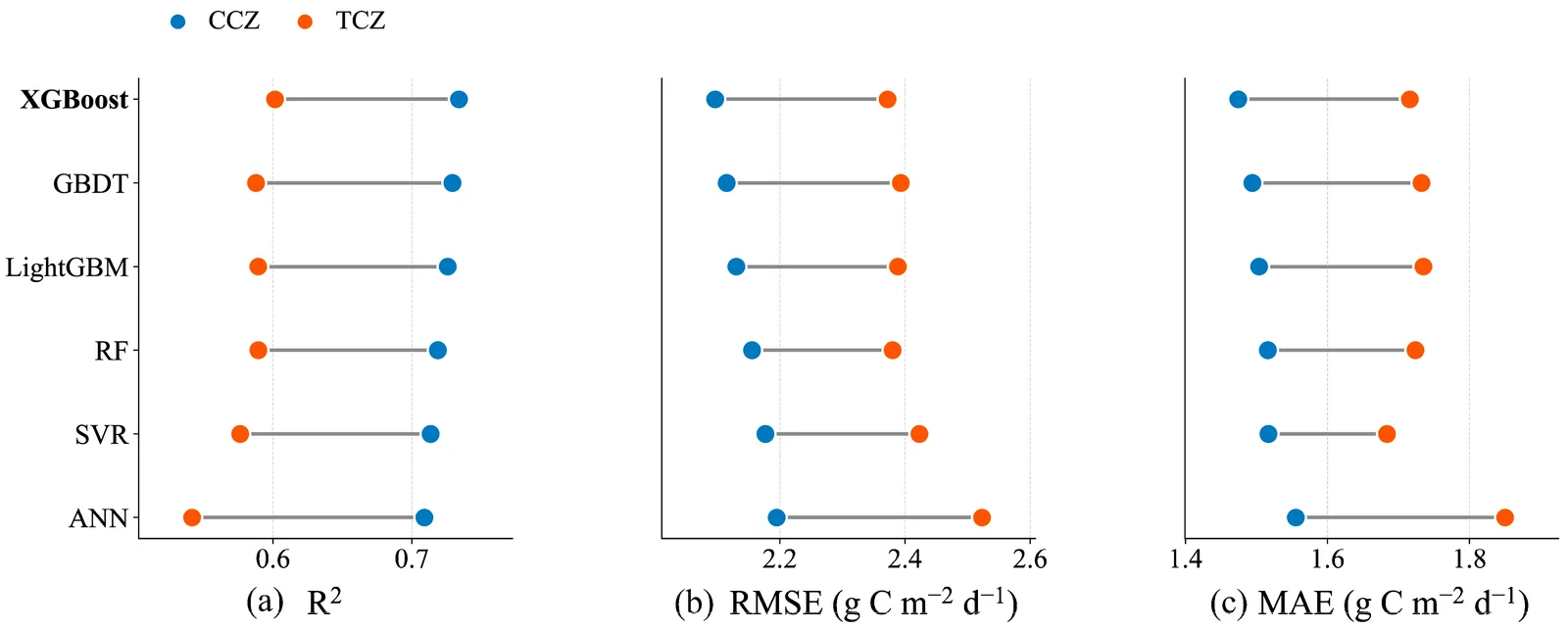
Predictive performance of six machine learning models in the TCZ (orange) and CCZ (blue) for daily GPP estimation. Note: (**a**) R^2^, (**b**) RMSE, and (**c**) MAE. Models are ranked in descending order of their mean R^2^. eXtreme gradient boosting, XGBoost; gradient boosting decision tree, GBDT; light gradient boosting machine, LightGBM; random forest, RF; support vector regression, SVR; and artificial neural networks, ANN.

As evidenced by five-fold cross-validation, the decision-tree-based ensemble learning models outperform support vector regression (SVR) and artificial neural networks (ANN) (Figure 1), with eXtreme gradient boosting (XGBoost) achieving the highest overall accuracy. Specifically, XGBoost explains 60.15% of daily GPP variation in TCZ forests, with RMSE = 2.37 g C m^−2^ d^−1^ and MAE = 1.72 g C m^−2^ d^−1^. By comparison, it accounts for 73.39% of daily GPP variation in CCZ forests, with RMSE = 2.10 g C m^−2^ d^−1^ and MAE = 1.47 g C m^−2^ d^−1^. Among all models, ANN performed the worst, with the lowest explanatory power for daily GPP variation (54.18% in TCZ and 70.90% in CCZ) and the highest RMSE and MAE. Collectively, these metrics show that XGBoost reliably captures daily GPP variation in both climate zones and was therefore chosen for further analysis.

### 2.2. Dominant Drivers of Forest Gross Primary Productivity

**Figure 2:**
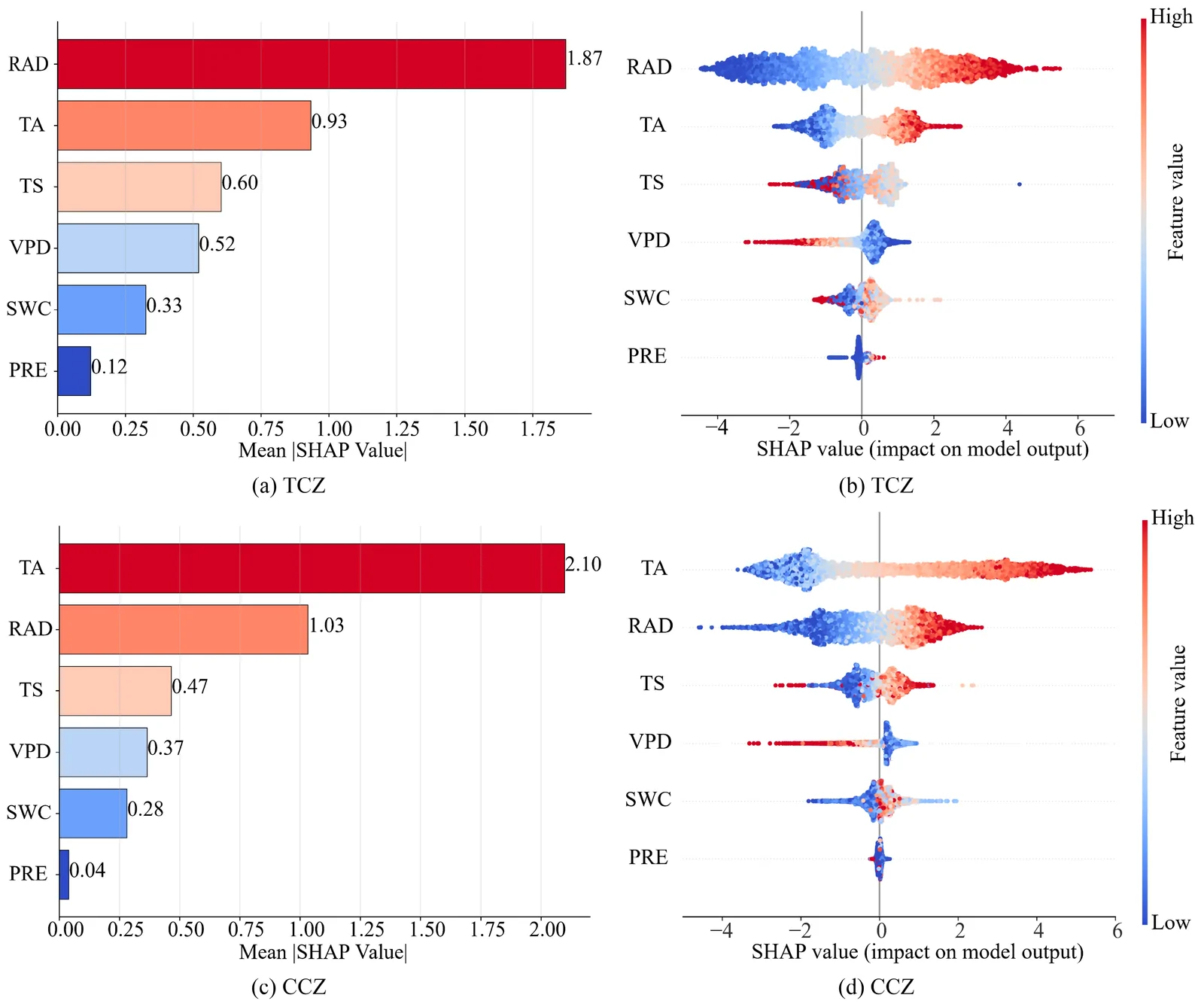
Global feature importance of the environmental factors affecting GPP using SHAP values. Note: (**a**,**b**) temperate climate zone; (**c**,**d**) cold climate zone; RAD, solar radiation; TA, air temperature; TS, soil temperature; VPD, vapor pressure deficit; SWC, soil water content; PRE, precipitation.

SHAP values of each environmental factor in all daily samples were quantified in both TCZ and CCZ forests. Among all environmental factors, solar radiation emerges as the dominant driver of forest GPP in TCZ, achieving a SHAP value of 1.87 g C m^−2^ d^−1^, whereas air temperature and soil temperature demonstrate secondary influences, with SHAP values of only 0.93 and 0.60 g C m^−2^ d^−1^, respectively (Figure 2a). By contrast, air temperature is the dominant driver of forest GPP in CCZ, followed by solar radiation and soil temperature, with SHAP values of 2.10, 1.03, and 0.47 g C m^−2^ d^−1^, respectively (Figure 2c). Compared with temperature and radiation, the contributions of PRE, SWC and VPD to GPP are limited in both climate zones. In addition, to mitigate uncertainties arising from geographic heterogeneity, we re-estimated the results at both the continental and site levels, which demonstrated robust findings (Figures S1 and S2).

The SHAP summary plots show the direction and strength of the impact of environmental factors on GPP (Figure 2b,d). Negative SHAP values flag an inhibitory effect on predicted GPP, whereas positive values mark a stimulatory effect. In TCZ forests, solar radiation shows a strong positive association with GPP, as evidenced by the right-skewed SHAP distribution with a long tail toward positive values, indicating that high radiation levels contribute substantially to the model’s GPP predictions (Figure 2b). In CCZ forests, the right-tailed extension of TA SHAP values reveals pronounced GPP stimulation as air temperatures rise (Figure 2d). Conversely, the SHAP distribution for RAD exhibits a pronounced left tail, with values becoming increasingly negative as radiation declines, underscoring its constraining effect on GPP under low-light conditions (Figure 2d).

### 2.3. Threshold Effect of Environmental Factors on Gross Primary Productivity

**Figure 3:**
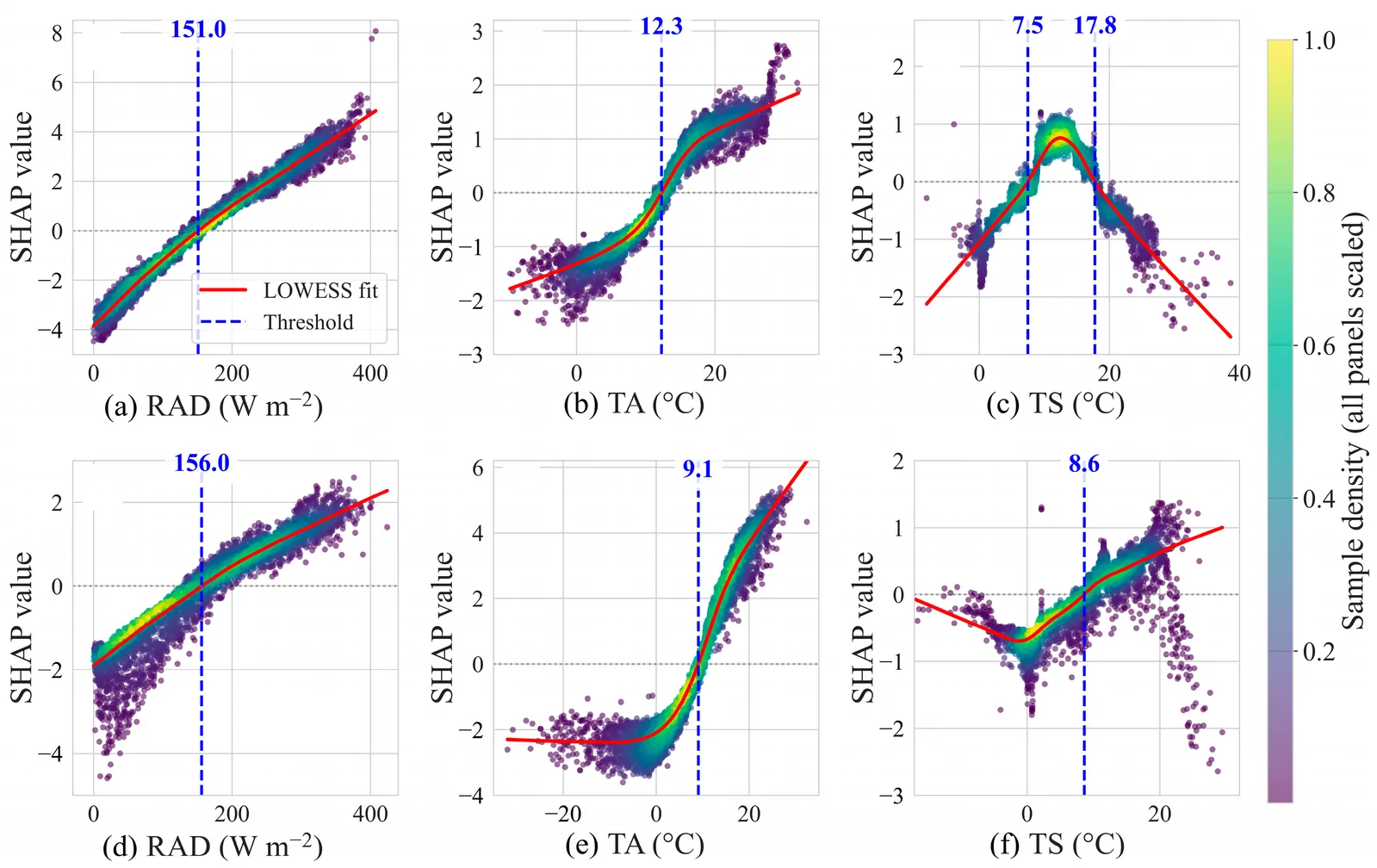
Nonlinear relationships between key environmental drivers and SHAP values. Note: (**a**–**c**) temperate climate zone; (**d**–**f**) cold climate zone; RAD, solar radiation; TA, air temperature; TS, soil temperature. Point color denotes sample density. Red lines are locally weighted regression (LOWESS) fits; blue dashed vertical lines mark thresholds where the effect shifts from negative to positive; in (**c**), the line at 17.8 °C marks the upper threshold beyond which the effect of TS on GPP becomes negative.

To investigate the nonlinear relationships between key driving factors and GPP, we examined the three factors with the largest mean |SHAP| values using dependence plots (Figure 3). These plots show how SHAP values vary with the magnitude of each factor, revealing critical thresholds where the effects on GPP shift (Table 1). In TCZ forests, a significant positive correlation was observed between RAD and its SHAP values (Figure 3a). When RAD exceeds 151.0 W m^−2^, it drives GPP upward, with higher RAD exerting a stronger positive effect. Conversely, below this threshold, RAD exhibits a negative effect. Air temperature is identified as the second most important driver of GPP in TCZ forests, with its SHAP value increasing monotonically with air temperature (Figure 3b). The threshold at which its effect on GPP shifts from negative to positive is approximately 12.3 °C. In contrast to the regulatory patterns of RAD and TA, TS exhibits a significant nonlinear regulatory effect (Figure 3c). At lower TS (<7.5 °C), its SHAP values are negative, indicating that low soil temperature suppresses GPP in TCZ forests. As TS rises above 7.5 °C, the SHAP values shift to positive, suggesting that moderate soil warming promotes GPP. The positive contribution of TS to GPP reaches its maximum at approximately 12.5 °C and declines at higher temperatures. Once TS exceeds 17.8 °C, this contribution becomes negative and TS begins to inhibit GPP (Figure 3c).

Similar to TCZ forests, CCZ forests exhibit a monotonically strengthening positive response of GPP to rising RAD (Figure 3d). The corresponding threshold at which the effect switches from limitation to promotion is 156.0 W m^−2^, slightly higher than that in the TCZ. Notably, when TA falls below 0 °C, it exhibits a pronounced inhibitory effect on GPP, but this inhibitory effect does not amplify with further declines in TA (Figure 3e). When TA exceeds 0 °C, its SHAP values show a positive correlation with TA and transition from inhibition to promotion at 9.1 °C. For TS, a distinct nonlinear pattern is observed, with a threshold of 8.6 °C for shifting from inhibition to promotion.

In addition, the trends observed in the SHAP analysis reveal that beyond the respective TA thresholds, GPP in the CCZ forest exhibits a markedly steeper sensitivity to TA than that in the TCZ forest (Figure 3b,e). In contrast, GPP in the TCZ forest is more sensitive to RAD (Figure 3a,d). Overall, these findings indicate that the CCZ forest GPP is constrained by a higher radiation threshold and a lower air temperature threshold, whereas the opposite patterns hold for the TCZ forest.

### 2.4. The Main and Interactive Effects of Environmental Factors

**Figure 4:**
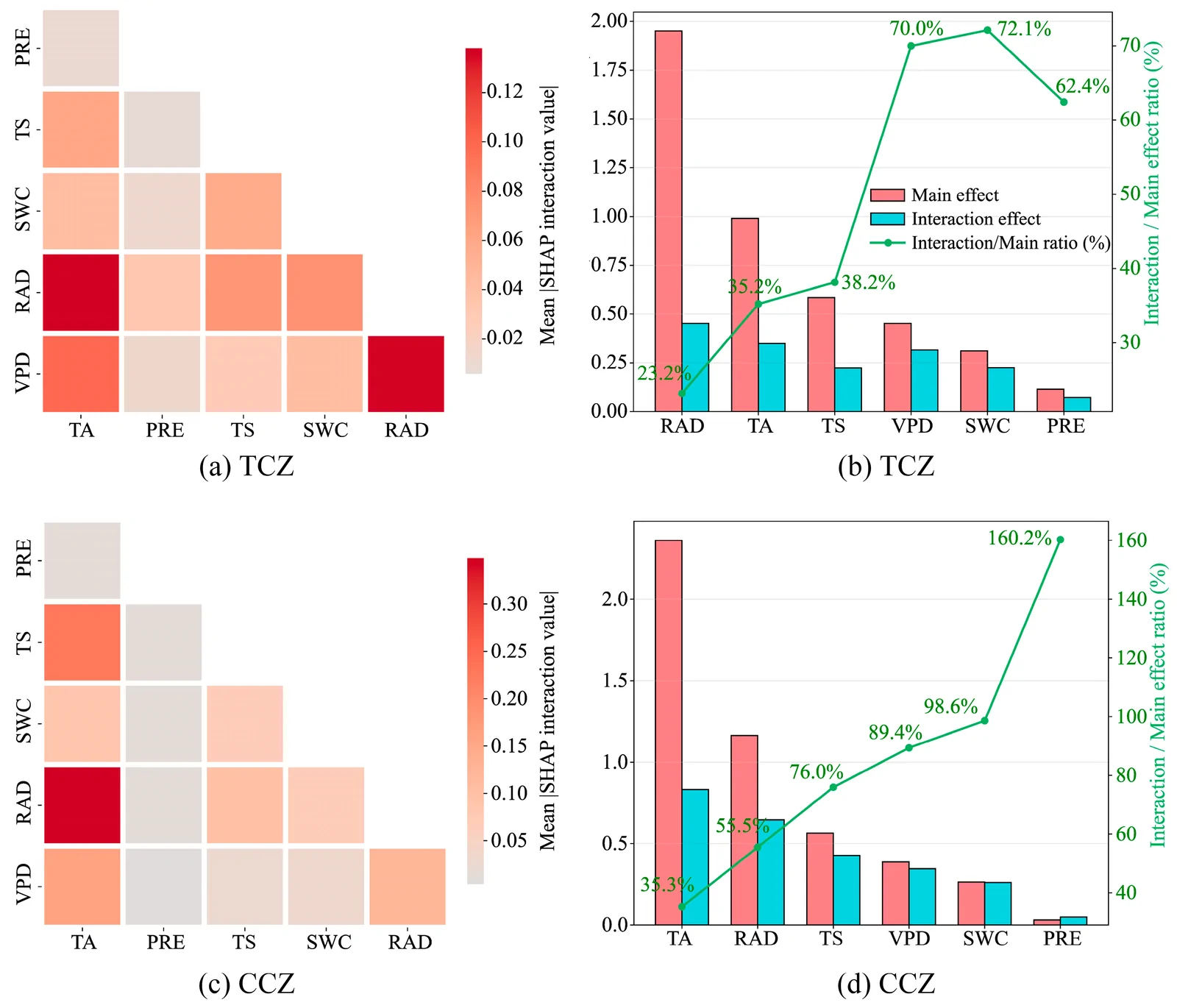
Pairwise interactions and main vs. total interaction effects for environmental drivers of GPP. Note: (**a**,**b**) temperate climate zone; (**c**,**d**) cold climate zone; RAD, solar radiation; TA, air temperature; TS, soil temperature; VPD, vapor pressure deficit; SWC, soil water content; PRE, precipitation.

To explore the regulatory mechanism of environmental factors on GPP, we separated the total effects of environmental factors into main and interactive effects based on the SHAP algorithm (Figure 4). For TCZ forest GPP (Figure 4a), the largest interaction effect is between RAD and TA (SHAP interaction value = 0.14 g C m^−2^ d^−1^), followed by the interaction between RAD and VPD (SHAP interaction value = 0.13 g C m^−2^ d^−1^). Similarly, for CCZ forest GPP (Figure 4c), a significant interaction is also observed between RAD and TA, with a SHAP interaction value of about 0.35 g C m^−2^ d^−1^. The interaction between TA and TS is equally prominent, yielding a SHAP interaction value of 0.23 g C m^−2^ d^−1^. The main effect of RAD is highest in TCZ forests (1.95 g C m^−2^ d^−1^), and that of TA is highest in CCZ forests (2.36 g C m^−2^ d^−1^; Figure 4b,d), confirming RAD and TA as the primary drivers in the two zones, respectively. Moreover, we observed that for environmental factors with high importance, the contribution from interactions is markedly lower than that from their main effects, whereas for less influential environmental factors, their interaction contribution is comparatively high (Figure 4b,d). We further calculated the ratio of interaction to main effect for each factor. In TCZ forests, the interaction contributions of the top three main factors, namely RAD, TA, and TS, account for 23.2%, 35.2%, and 28.2% of their corresponding main effect contributions, respectively, while the ratios for other variables range from 62.4% to 72.1%. In CCZ forests, the top three drivers (TA, RAD, and TS) exhibit interaction/main-effect ratios of 35.3%, 55.5%, and 76.0%, respectively, while the ratios for VPD, SWC and PRE increase to 89.4%, 98.6%, and 160.2%. These results show that interactions between dominant factors and other environmental factors contribute far less to GPP variation than their individual effects. Although the individual contribution of non-dominant factors is limited, their interactions with other factors make a non-negligible contribution.

**Figure 5:**
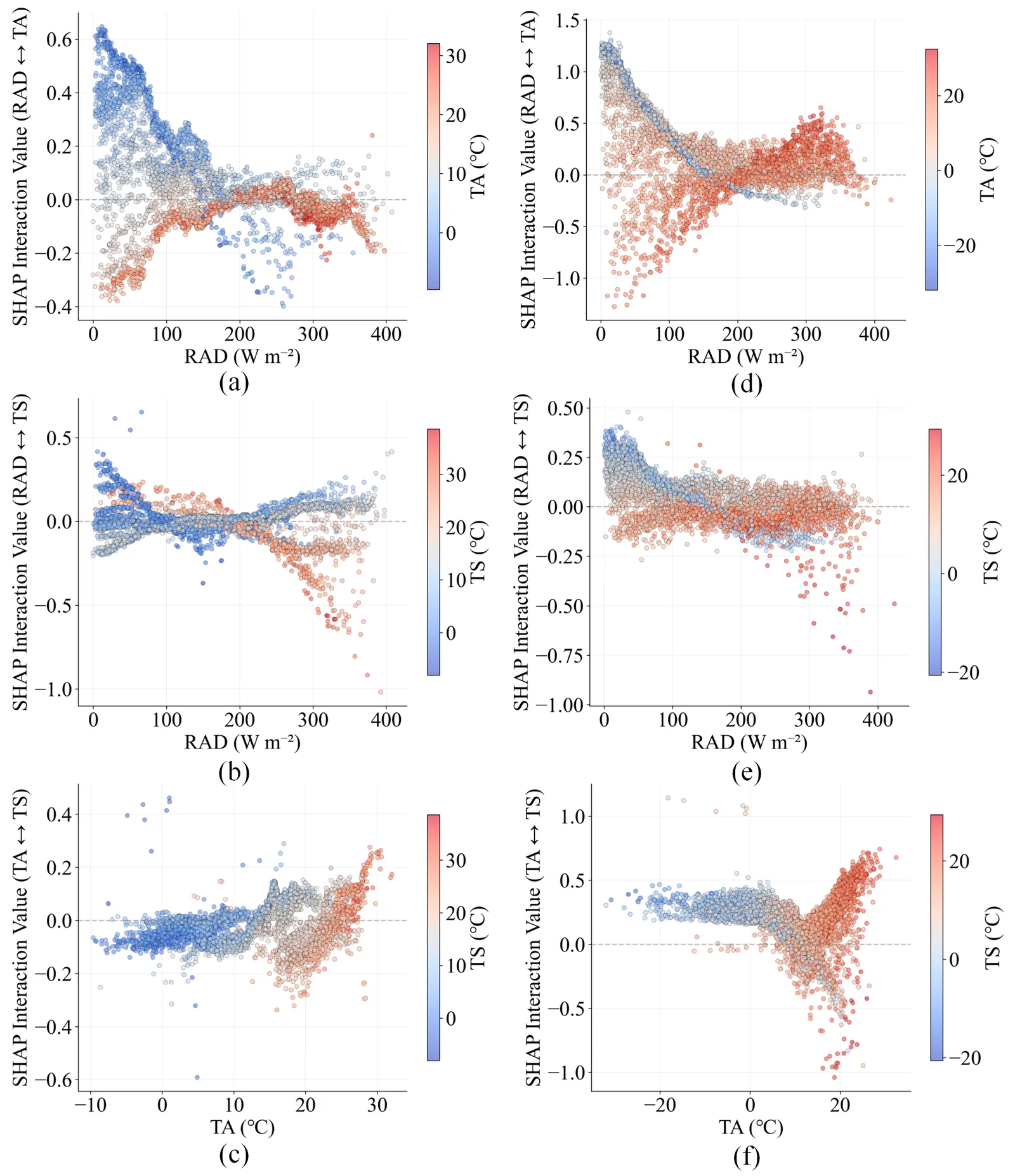
The interaction relationships between the key variables affecting GPP. Note: (**a**–**c**) temperate climate zone; (**d**–**f**) cold climate zone; RAD, solar radiation; TA, air temperature; TS, soil temperature.

In this case, the complex interaction patterns among environmental variables were identified using the SHAP interaction plot (Figure 5). The vertical dispersion in the plot reflects the strength of interactions, with wider dispersion indicating stronger interactions. The analysis revealed pronounced nonlinearities, with the contribution of each factor strongly conditional on the level of its interacting covariate. In TCZ forests, RAD exhibits pronounced interactive effects with both TA and TS (Figure 5a,b), underscoring that the GPP response to radiation is closely modulated by temperature conditions. For instance, when RAD is below approximately 190 W m^−2^, rising TA progressively attenuates the interaction values between RAD and TA. Once RAD exceeds 190 W m^−2^, this trend reverses (Figure 5a). This interaction pattern indicates that GPP only benefits from rising temperature once plants are no longer constrained by radiation availability. Furthermore, when the air temperature is at a low level (blue dots in Figure 5a), the interactions between RAD and TA decrease as RAD increases, implying that the increase in light intensity under cooler conditions suppresses GPP. Notably, under high radiation conditions (>220 W m^−2^), increasing TS corresponds to lower SHAP interaction values, highlighting a negative interactive effect between TS and RAD (Figure 5b). Given the close relationship between TA and TS, a notable interaction also exists between these two variables (Figure 5c). When TS is at low levels (blue dots in Figure 5c), an increase in TA does not appear to alleviate the negative effect on GPP. In contrast, when TS is at high levels (brown dots in Figure 5c), the two factors exhibit a positive interactive effect on GPP.

In CCZ forests, TA emerges as the dominant environmental driver of GPP, and the pronounced vertical dispersion in Figure 5d underscores its strong interaction with RAD. When TA falls below approximately 0 °C (blue dots in Figure 5d), rising RAD progressively reduces the SHAP interaction values. Conversely, under high TA (brown dots in Figure 5d), higher RAD is associated with increased SHAP interaction values. Additionally, under low radiation conditions (<160 W m^−2^), rising TA lowers the SHAP interaction value, indicating suppression of GPP. In contrast, under high radiation, rising TA corresponds to higher interaction values. Notably, the coupling between RAD and TA exerts a stronger synergistic effect on GPP under low-temperature and low-light conditions in the CCZ, as evidenced by a substantially wider range of interaction values (Figure 5a,d). However, as RAD intensity increases, this synergistic interaction rapidly attenuates to near zero, indicating that under strong light conditions, the effect of temperature on GPP becomes largely independent of radiation. Owing to the significant correlation between TA and TS, the interaction pattern between TS and RAD resembles that between TA and RAD (Figure 5e). The interaction pattern between TA and TS in CCZ forests is similar to that in TCZ, where both variables show a positive interactive association with GPP only when TS is at a high level (Figure 5f). That is, high TS conditions are required for positive interactions between TA and TS in both zones; however, the magnitude and consistency of this effect appear greater in CCZ forests, reflecting stronger temperature constraints on GPP in colder environments (Figure 5c,f).

## 3. Discussion

### 3.1. Relative Importance Analysis

The substantial environmental differences between the temperate and cold climate zones result in marked differences in the GPP response to environmental change. Our results show that solar radiation and air temperature are the primary environmental drivers of daily GPP variation in TCZ and CCZ forests, respectively (Figure 2). This agrees with evidence at the site level that radiation dominates daily GPP variation in warm, humid forests [10,12,24]. A recent analysis of 98 site years across 14 European evergreen needleleaf forests reports that radiation dominates net ecosystem productivity at the warmer southern sites and that air temperature dominates at the colder sites [25], a contrast within the same continent that parallels the regional comparison made here (Figure S1). A site-level analysis of three warm temperate sites in China (CN-DHS, CN-HTF and CN-QYZ) further confirmed that RAD governs daily GPP variation in TCZ forests (Figure S2). In CCZ forests, daily GPP variation is driven primarily by air temperature, which may be attributable to the relatively cold environment, where the mean daily air temperature is only 4.96 °C (Table 2). This temperature dominance is also supported by recent regional evidence. In the mountains of central China, temperature was identified as the dominant and persistent driver of GPP with the same explainable machine learning design as the present study [26]. Across 108 North American eddy covariance sites, air temperature was likewise reported as the main productivity driver in wet ecosystems [27]. In boreal and cold temperate regions, temperature was found to primarily constrain the enhancement of forest GPP by CO_2_ fertilization [28]. Structural equation modeling and correlation analyses further support these dominant roles (Figures S3 and S4).

Discrepancies in the dominant drivers of forest GPP among climatic zones reflect contrasting climatic sensitivities [29]. TCZ forests are characterized by abundant heat and moisture (Table 2), which jointly supply optimal hydrothermal resources for photosynthetic activity. Incident radiation directly powers the light reactions, and carbon assimilation increases correspondingly with irradiance (Figure 3a). Furthermore, Figure 3a shows that GPP in TCZ forests is more sensitive to changes in RAD than in CCZ forests. Studies have also shown that the dense canopies of TCZ forests occupy substantial vertical space, intensifying competition for limited light resources [30]. Compared with TCZ forests, CCZ forests are characterized by markedly cooler air temperatures (Table 2). Growing-season temperatures typically remain at or below the thermal optimum for photosynthesis in CCZ forests [31], implying that carbon fixation in these ecosystems is chronically temperature-limited. Extreme low temperatures depress Rubisco catalytic efficiency and destabilize thylakoid membranes, thereby constraining the capacity of radiation to enhance GPP [32,33].

Forest types differ markedly between the two climate zones (Table 3). The TCZ sites are dominated by evergreen and deciduous broadleaf forests and by mixed broadleaf and conifer stands, whereas the CCZ sites are dominated by coniferous and boreal forests. This contrast is not incidental to the design, because forest composition and species richness covary with the climatic gradient that separates the two zones, and both are known to modify the strength of the environmental controls on productivity [34,35]. Local richness places this contrast on a quantitative footing at the scale of the stands themselves. When all sites are pooled, richness is higher in the temperate zone (median 4.6 versus 3.9 species per hectare), but the difference is unevenly distributed. It arises almost entirely from the East Asian and Australian sites (28.8 and 30.6 species per hectare) and is negligible within Europe (4.3 versus 3.9) and North America (3.0 versus 3.1). Local richness therefore does not distinguish the two zones once the region is held constant, whereas the contrast in the dominant driver persists within each climate zone (Table 3). Two qualifications apply to the ranking of driving factors reported here. First, the ranking describes the aggregate behavior of the forest types actually sampled and should not be extrapolated to stands outside the range of composition and richness represented in this study. Second, because the ranking is preserved when driver importance is estimated separately within each region (Table 3, Figure S1), it cannot be attributed to the compositional imbalance between the zones, although composition remains a covarying rather than a controlled factor.

In the context of a multivariate data-driven model, the prediction is not exclusively dependent on a subset of features [17]. Although SWC, VPD and PRE are individually ranked low in feature importance, they strongly modulate model performance through interactions with other variables (Figure 4), highlighting the significance of their interdependence.

### 3.2. Significance and Nonlinear Effects of Environmental Factors

Environmental control of GPP is generally nonlinear, with effects differing markedly before and after critical thresholds [12]. Across both TCZ and CCZ forest ecosystems, GPP responds nonlinearly to rising RAD (Figure 3a,d). SHAP values tracking RAD levels follow the classical vegetation light response curve, suggesting that increased radiation rapidly enhances light-use efficiency under radiation-limited conditions [36]. Across the RAD range covered by observations, SHAP values increase monotonically in both zones without saturation signals (Figure 3a,d). Notably, the RAD threshold at which the effect on GPP switches from limitation to promotion is lower in TCZ forests than in CCZ forests (Figure 3), implying that temperate forest canopies have superior light-use efficiency and can translate weaker radiation increments into GPP gains [37]. Temperate forests typically feature complex, multi-layered canopies that create strong within-canopy light gradients [38]. Their shade-adapted leaves exhibit low light saturation points and high light utilization efficiency [39,40], making the photosynthetic capacity of TCZ forests primarily limited by the availability of solar radiation. In contrast, the higher GPP response threshold to RAD in CCZ forests indicates that light-use efficiency is constrained by multiple factors. For example, low temperatures inhibit photosynthetic enzyme activity, requiring higher RAD to compensate for thermal deficiency [41].

Temperature directly affects forest GPP dynamics through multifaceted regulation of photosynthetic enzyme activity [42], water-use efficiency [43], and light-use efficiency [44]. These underlying mechanisms account for the observed threshold divergence in temperature responses of forest GPP across climatic zones. Our results reveal that the limiting threshold of air temperature for CCZ forest GPP is lower than that for TCZ forest GPP (Figure 3). Research shows that CCZ forest leaves exhibit high temperature plasticity by adjusting the activity and abundance of key photosynthetic enzymes [45]. As soon as spring air temperatures rise above a low threshold, trees rapidly initiate growth processes such as budburst and leaf-out to make full use of the short favorable growing window [46,47]. TCZ forests, by contrast, buffer warming by enhancing heat tolerance rather than adjusting photosynthetic parameters [45]. Accordingly, the photosynthetic system of TCZ forests shows a limited capacity to adjust in response to air temperature variations, which explains why its temperature threshold for initiating substantial photosynthesis and growth is higher than that of the CCZ forest (Figure 3). It is noteworthy that the threshold at which soil temperature transitions from inhibition to promotion is higher in CCZ forests, approaching the air temperature threshold of this climate zone. This phenomenon may be attributable to the stronger coupling between air and soil temperatures in such regions [48]. In addition, the higher temperature sensitivity of soil organic matter decomposition accelerates nutrient release as soil temperature rises [49,50]. In contrast, in TCZ, thermal conditions no longer serve as the primary limiting factor. Instead, rising soil temperature intensifies water evaporation and plant transpiration, inducing drought stress and consequently weakening the positive effect of warming on GPP [51]. However, once soil temperature exceeds the upper threshold, excessive heat may affect GPP through two pathways: direct heat stress, such as damage to photosynthetic enzymes and photosystem II or increased photorespiration [52], and indirect drought effects, such as accelerated soil desiccation (i.e., the pronounced drying of the soil profile under high evaporative demand) and reduced plant water availability [53]. This soil drought may coincide with atmospheric water deficits during the growing season, compounding stress and reducing productivity [7].

The air temperature thresholds identified here (9.1 °C in the CCZ and 12.3 °C in the TCZ) exceed the 3 to 4 °C air temperature reported to trigger the recovery of stand-level photosynthesis in boreal evergreen forests [54]. It should be emphasized that, although photosynthesis begins to recover once air temperature exceeds 3 to 4 °C, the photosynthetic rate remains severely constrained at such low temperatures. Our results indicate that soil temperature contributes positively to GPP up to an upper threshold of 17.8 °C in the TCZ, above which its contribution becomes negative (Figure 3c), and that this positive contribution reaches its maximum at approximately 12.5 °C. This upper threshold coincides with in situ observations of a temperate hardwood forest, in which root elongation of mature black walnut peaked at soil temperatures of 17 to 19 °C and declined beyond them, indicating that soil warming above this window no longer enhances root growth [55]. In contrast, no comparable upper threshold was identified in the CCZ, where soil temperature acted predominantly as a limiting factor and suppressed GPP only in a subset of observations with soil temperatures above approximately 20 °C (Figure 3f). This pattern is consistent with the response of photosynthesis to soil temperature in boreal tree species, in which photosynthesis declines once soil temperature exceeds the range of 20.9 to 22.0 °C [56]. However, the apparent suppression at the warm end of the CCZ range may partly reflect covariation between soil temperature and atmospheric drought rather than the direct effect of soil temperature itself, because dry soils warm disproportionately as more of the absorbed energy is released as sensible heat, which in turn increases the atmospheric demand for water [57]. Our paired-zone design demonstrates that these thresholds are not universal constants but shift systematically with climatic context (Table 1). Such spatially varying shifts caution against transferring threshold values between climate zones and underscore the need for zone-specific calibration in process-based models.

### 3.3. Complex Interaction Effects Between the Key Drivers of Gross Primary Productivity

The nonlinear influence of environmental factors on GPP arises not only from variations in their individual magnitudes but also from their interactions. By isolating main and interactive effects, our results show that the main effect contribution of the dominant factor is significantly greater than its interaction contribution with other factors in both TCZ and CCZ forests (Figure 4b,d). Similarly, Zhou, Yue, Wang, Tian, Lu, Zhu and Cao [10] found that environmental factors regulate GPP variation primarily through main effects rather than interactive effects. Previous studies based on path analysis have also shown that the direct influence of environmental variables on GPP variation outweighs their indirect influence [6,11]. This pattern is consistent with the expectation derived from Liebig’s law of the minimum [58], which states that plant growth is ultimately constrained by the single most limiting resource. For TCZ and CCZ forests, radiation and temperature act as the primary limiting factors on productivity, respectively [11,59]. When an environmental factor is strongly limiting, changes in other factors have relatively little additional impact on growth, so the interaction contribution remains minor compared to the main effect of the limiting factor. Once the limiting factor is alleviated, however, interactive effects are expected to increase, as supported by our interaction analysis (Figure 5).

Although the main effects of radiation and temperature account for the majority of GPP dynamics in TCZ and CCZ forests, their interactions exert a significant complementary control. Our study reveals that the effect of temperature on GPP in TCZ forests is light-dependent. Elevated temperatures suppress GPP under low-light conditions but enhance it under high light availability (Figure 5a,d). This phenomenon may stem from the limited light reaction rate under low-light conditions, where the energy produced cannot keep pace with the accelerating demand of dark reactions [60]. Although warming stimulates photosynthesis enzymes, the extra energy burden of increased photorespiration and basal metabolism simultaneously erodes light-use efficiency [61]. Under comparatively high RAD, the photoreaction provides ample reductant, and the same temperature increment accelerates carboxylation without incurring an energy shortfall, thereby yielding a net gain in carbon assimilation [62]. Notably, when TCZ forests experience simultaneously high radiation and high temperature, the interaction between the two factors suppresses GPP (Figure 5a). This suppression is likely attributed to the intensified photoinhibition caused by elevated temperatures [4].

Three features of the RAD response in the CCZ deserve emphasis. First, although TA ranks first, radiation remains a strong secondary limitation. The SHAP dependence on RAD rises monotonically without saturation (Figure 3d), and the zone mean RAD of 142.26 W m^−2^ (Table 2) lies below the 156.0 W m^−2^ threshold (Table 1), so a substantial fraction of daily observations remains on the limitation side of the light response. Second, the interaction between RAD and TA is the largest pairwise interaction in the CCZ (0.35 g C m^−2^ d^−1^, Figure 4c), roughly two and a half times its TCZ counterpart (0.14 g C m^−2^ d^−1^), and its sign is conditional on the thermal state. Rising RAD suppresses GPP when TA falls below about 0 °C, whereas under warmer conditions higher RAD enhances GPP (Figure 5d,e). Correspondingly, the interaction-to-main-effect ratio of RAD reaches 55.5% in the CCZ against 23.2% in the TCZ (Figure 4b,d), confirming that in cold forests the effect of radiation operates to a much larger extent through temperature-dependent pathways. Third, this synergy is most pronounced under simultaneous low temperature and low radiation and attenuates rapidly to near zero as RAD increases, indicating that once light ceases to be limiting, the effect of temperature becomes largely independent of radiation (Figure 5a,d). Physiologically, the higher RAD threshold of the CCZ reflects the depressive effect of low temperatures on Rubisco catalytic efficiency and thylakoid stability, so a stronger photon flux is required before radiation turns net-promoting [32,33]. The suppression of GPP by radiation under cold conditions further reflects restricted root hydraulic conductance and nutrient uptake, which combine with radiation-induced increases in leaf temperature and transpiration demand to close stomata and trigger photoinhibition [63,64,65], whereas under warmer conditions water and nutrient supply keep pace with the radiation-driven demand [66]. For management, this implies that in the CCZ, measures that increase light availability alone, such as thinning or gap creation, are unlikely to raise GPP unless the thermal constraint is relieved at the same time.

### 3.4. Implications and Future Perspectives

The quantitative thresholds identified here offer actionable guidance for sustaining forest carbon sinks under climate change. First, the thresholds in Table 1 serve as early warnings of productivity decline. In the TCZ, radiation below 151.0 W m^−2^ or air temperature below 12.3 °C suppresses GPP, favoring light-enhancing practices such as gap creation or reduced stand density during overcast or cooler periods. In the CCZ, the 9.1 °C threshold supports promoting tree species with lower thermal requirements for photosynthesis to buffer productivity losses at higher elevations or in cooler years. At lower temperatures, such as the 9.1 °C threshold identified for the CCZ in this study, carboxylation capacity, electron transport and photosystem II efficiency are directly temperature-limited [32,33,42], so each additional degree produces a large marginal gain in GPP, whereas above the threshold the gain flattens as radiation and water become co-limiting. The ecosystem-level temperature optimum of photosynthesis—about 18 °C [67]—lies far above the mean growing season temperature of the CCZ forests (Table 2), which places this zone on the steep cold limb of the temperature response, where warming is most effective at raising carbon input. The carbon stock depends on the balance between the carbon input of photosynthesis and the carbon losses through autotrophic and heterotrophic respiration, as well as on the carbon residence time. Respiration continues to rise with temperature above the photosynthetic optimum [67], and long-term soil warming experiments show that the resulting respiratory losses can offset much of the gain in input [68]. A forest in which temperature controls the input can therefore gain carbon early in the growing season and lose it later. The thresholds in Table 1 are therefore transition points on the input side of the carbon balance rather than evidence of a larger carbon stock. Second, the interaction-to-main-effect ratios in Figure 4 show that non-dominant factors, including vapor pressure deficit, soil water, and precipitation, influence GPP mainly through interactions with dominant drivers. Thus, actions targeting these secondary factors alone, such as irrigation, have limited impact unless paired with measures addressing the primary limiting factor. Nature-based solutions should therefore adopt zone-specific, multi-factor strategies. Finally, these controls are not equally important everywhere. In tropical forests, where temperature is rarely limiting and radiation is abundant, vapor pressure deficit and soil moisture dominate GPP variability [69,70,71]. The radiation and temperature thresholds identified here would therefore be expected to shift or even disappear in those systems. Our results are thus best interpreted as a description of the temperate and cold forest domain, in which energy supply rather than water supply is the first-order constraint.

While offering valuable insights, this study is subject to several limitations. First, GPP responds to environmental drivers in ways that vary markedly across temporal scales [10], whereas our study was restricted to the daily scale. Future work will exploit weekly, monthly, and annual datasets to test whether the thresholds and interaction patterns change at longer scales. Second, CO_2_ concentration was not considered because daily CO_2_ variability is small and its fertilization effect is overshadowed by meteorological fluctuations; neglecting it does not introduce significant uncertainty [10,13]. Finally, this study did not explicitly account for the diversity of forest types (e.g., evergreen vs. deciduous, broadleaf vs. coniferous) within the temperate and cold zones. Future efforts will incorporate eddy-covariance records from other regional networks, climate zones, and forest types to corroborate our findings.

## 4. Materials and Methods

### 4.1. Observation Data

**Figure 6:**
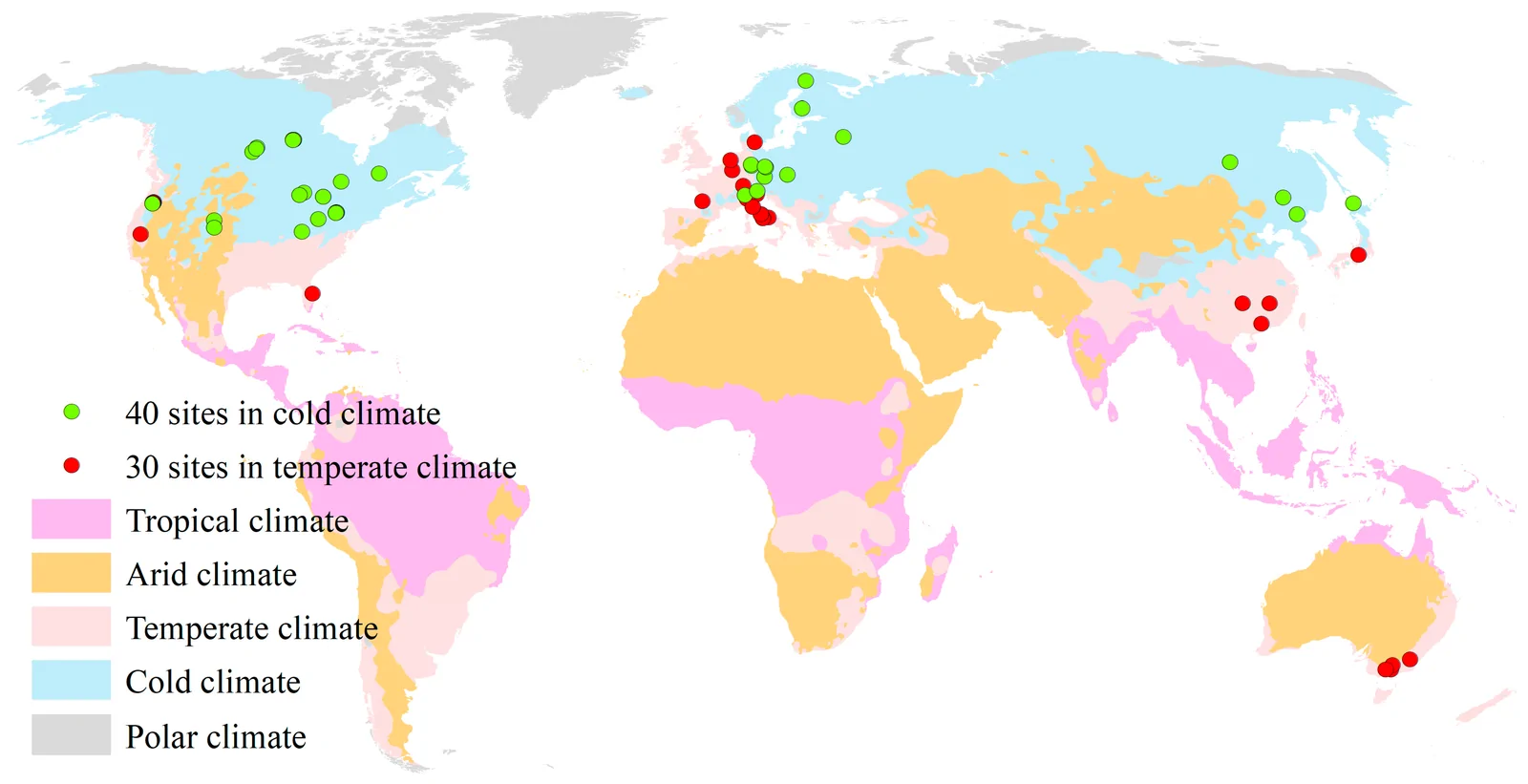
Geographic locations of the 70 flux tower sites used in this study. The climate zone data are derived from the Köppen–Geiger climate classification [23].

Daily meteorological data were used to drive ML models for simulating forest GPP. The driving variables include TA (°C), PRE (mm), RAD (W m^−2^), VPD (hPa), TS (°C), and SWC (%), with GPP serving as the target variable. These data are sourced from publicly available databases, including FLUXNET (https://fluxnet.org/, accessed on 5 October 2026), ChinaFLUX (http://www.chinaflux.org/, accessed on 5 October 2026) and the National Ecosystem Science Data Center (https://www.nesdc.org.cn/, accessed on 5 October 2026). Since soil temperature and water content were observed only at a subset of flux sites, we restricted our analysis to sites with all driving variables (Figure 6), ultimately selecting 30 forest flux sites in the TCZ and 40 in the CCZ according to the Köppen–Geiger climate classification [23]. Detailed site information is presented in Table S1. Table 2 summarizes the climatic characteristics of the two zones derived from site-based observations. Daily GPP was aggregated from original half-hourly CO_2_ flux measurements obtained via the eddy covariance technique, following standard quality control and gap-filling protocols [72,73]. For the forest sites in the CCZ, only days with GPP > 0 were retained for analysis, thereby focusing on the active growing season.

To examine whether forests in the same continental region but in different climate zones differ in their GPP response to climate, we further assigned the 70 sites to four regions, East Asia, Europe, North America and Australia, according to their geographical distribution (Figure 6, Table 3). Dominant forest types are compiled from national forest inventories and regional syntheses: GlobalTreeSearch for East Asia and Australia (https://tools.bgci.org/global_tree_search.php, accessed on 5 October 2026); the European Red List of Trees for Europe [74]; and the standardized checklist of US native tree species for North America [75]. Tree species richness is the mapped number of tree species per hectare at the coordinates of each tower, extracted from the global forest tree species richness map of Liang et al. [76] at 0.025° (about 2.5 km) resolution.

### 4.2. Methods

#### 4.2.1. Machine Learning Models

Decision-tree-based ensemble learning models have demonstrated strong performance in simulating carbon cycle dynamics using tabular data [16,20]. In this study, we selected four representative algorithms to estimate GPP, including random forest (RF), XGBoost, gradient boosting decision tree (GBDT), and light gradient boosting machine (LightGBM). While RF reduces variance by building a large ensemble of independent trees and aggregating their predictions, the other three models adopt a gradient boosting strategy, iteratively minimizing bias at each step to improve overall prediction accuracy. Taking XGBoost as an example, the model iteratively adds shallow decision trees that learn the residuals of their predecessors, and the final estimate is the weighted sum of all trees [77]. An embedded regularization term in the objective function constrains excessive complexity, curbing over-fitting far more effectively than conventional gradient-boosting machines [13]. This approach affords a finer local approximation of the loss function and accelerates convergence to optimal tree structures. In addition to these tree-based approaches, we also evaluated ANN and SVR to benchmark predictive accuracy and identify the optimal model for subsequent analysis.

All models were trained and validated using five-fold cross-validation at the daily sample level to prevent data leakage between training and validation. Hyperparameter tuning for each model was conducted using a random search strategy [78], and the resulting optimal parameter sets, along with their search spaces, are provided in Table S2. Model performance was evaluated using R^2^, RMSE, and MAE. All models were constructed and trained in Python 3.11.7 (Anaconda Inc., Austin, TX, USA).

#### 4.2.2. SHapley Additive exPlanations Method

Conventional ML models are inadequate for providing rational explanations for individual predictions and model decisions. To uncover how environmental factors shape variations in forest GPP and to quantify their individual and interactive effects, we employed the SHAP method based on TreeExplainer to deliver local and global interpretations of XGBoost predictions [79]. The SHAP method treats all features as contributors and quantifies the marginal contribution of a feature by comparing model predictions with and without that feature. The average marginal contribution across all possible subsets then defines its SHAP value.

By decomposing any prediction into the sum of individual feature contributions, SHAP reveals whether each variable pushes the output upward or downward and has become a valuable tool for local interpretation and global importance ranking in ecosystem analyses [13,16]. The calculation of SHAP values for each input variable is given by the following formula [80].

(1) $$\emptyset_{i}=\sum_{S\subseteq N\backslash \{i\}}\frac{\left|S\right|!\left(\left|N\right|-\left|S\right|-1\right)!}{\left|N\right|!}\left[f_{S\cup \left\{i\right\}}\left(x_{S\cup \left\{i\right\}}\right)-f_{S}\left(x_{S}\right)\right]$$

where ∅*_i_* represents the SHAP value of feature *i*, *N* is the total number of features, *S* represents any subset of features that does not include feature *i*, |*S*| is the feature number in subset *S*, *f_S_*(*x_S_*) is the contribution of feature subset *S* to model prediction, *f_S_*_∪{*i*}_(*x_S_*_∪{*i*}_) represents the contribution of feature set *S* ∪ {*i*} containing feature *i* to model prediction, and *f_S_*_∪{*i*}_(*x_S_*_∪{*i*}_) − *f_S_*(*x_S_*) represents the marginal contribution of feature *i* in subset *S*. Global feature importance is obtained by averaging the absolute SHAP values across all samples:

(2) $$I_{\mathrm{SHAP}}^{i}=\frac{1}{n}\sum_{k=1}^{n}\left|\emptyset_{i}^{k}\right|$$

where $I_{\mathrm{SHAP}}^{i}$ represents the global importance of feature *i*, $\emptyset_{i}^{k}$ stands for the SHAP value of feature *i* for the *k^th^* sample, and *n* is the number of samples.

The SHAP framework offers a theoretical approach to quantifying the independent and interactive effects of features [79]. The independent effect of a feature is represented by its main effect value, which reflects its isolated impact on the model output without interacting with other features. In turn, the interactive effect value captures the extra gain or loss that emerges when paired features act together. For each feature, its SHAP value can be divided into main and interactive effects [10]:

(3) $$\emptyset_{i}=\;\emptyset_{i,i}+\sum_{i\neq j}\emptyset_{i,j}$$

where ∅*_i_*_,*i*_ is the main effect of feature *i*, ∅*_i_*_,*j*_ represents the interaction effects between features *i* and *j*. The SHAP value ∅*_i_* of feature *i* equals the sum of its main effect plus all pairwise interactions with every other feature.

#### 4.2.3. Threshold Identification of Environmental Drivers

To investigate the pattern of response shifts in GPP under varying environmental conditions, we incorporated the threshold effect concept [12,13]. In this study, a threshold is defined as the value of an environmental variable at which its SHAP value crosses the zero line, marking a shift in its effect on GPP from suppressive to stimulatory or vice versa. For each key driver, SHAP values were plotted against the variable magnitude across all daily observations, and a locally weighted regression (LOWESS) curve was fitted to characterize the nonlinear response trend without assuming a predefined functional form. The smoothing span was set to 0.4, and two robust reweighting iterations were applied to reduce the influence of outliers during fitting. To further reduce the leverage of extreme observations, samples deviating from the lower or upper quartile by more than three times the interquartile range were excluded prior to fitting. Thresholds were then identified as the intersections between the LOWESS curve and the zero SHAP line.

To quantify the uncertainty of the identified thresholds, we employed a site-level block bootstrap. Daily observations were grouped by flux site, and sites were resampled with replacement 1000 times. For each replicate, the LOWESS curve was refitted and the threshold was re-estimated using the identical procedure. The 2.5th and 97.5th percentiles of the bootstrap distribution were taken as the 95% confidence interval. Site-level resampling preserves within-site dependence and yields more conservative uncertainty estimates than resampling individual days. Threshold sensitivity to the smoothing span was examined by repeating the analysis with spans from 0.3 to 0.7.

#### 4.2.4. Structural Equation Modeling

To further explore the direct and indirect effects of environmental variables on GPP, we employed structural equation modeling (SEM) to quantify these pathways. Path coefficients of the SEM were estimated using maximum likelihood with robust standard errors, as implemented in the “lavaan” package (version 0.6-20) in R 4.5.1 [81]. Standardized path coefficients are reported to compare effect sizes across variables. Model fit was assessed using the comparative fit index (CFI), Tucker–Lewis index (TLI), and standardized root mean square residual (SRMR). CFI and TLI values close to 1, together with SRMR values close to 0, indicate a well-fitting model.

## 5. Conclusions

In this study, we investigated the impact mechanisms of environmental factors on daily forest GPP variations by integrating the XGBoost model with the SHAP algorithm across temperate and cold climate zones. Our results indicate that solar radiation and air temperature are the dominant factors influencing forest GPP in temperate and cold climate zones, respectively. Forest GPP shows a lower radiation but higher air temperature limitation threshold in the temperate zone and the reverse in the cold zone. The individual effect of the dominant factor on forest GPP significantly outweighs its interactive effects with other environmental factors, whereas non-dominant factors play an important regulatory role through interactions. The coupling between RAD and TA is stronger in the cold climate zone than in the temperate zone, with pronounced synergistic effects under low-temperature and low-light conditions. The identified thresholds and interactions provide robust benchmarks for refining and parameterizing ecosystem process-based models and have implications for forest management and climate policy.

## Figures and Tables

**Table 1:** Thresholds of the top three environmental drivers of GPP.

	RAD (W m^−2^)		TA (°C)		TS (°C)	
	Threshold	95% CI	Threshold	95% CI	Threshold	95% CI
TCZ	151.0	[149.6, 152.5]	12.3	[12.2, 12.4]	7.5	[7.3, 7.6]
CCZ	156.0	[153.7, 158.1]	9.1	[9.0, 9.2]	8.6	[8.5, 8.8]

Note: TCZ: temperate climate zone; CCZ: cold climate zone; RAD: solar radiation; TA: air temperature; TS: soil temperature. Values in square brackets denote 95% confidence intervals (CI) derived from 1000 site-level bootstrap resamples.

**Table 2:** Climate characteristics of temperate and cold climate zones.

	TA (°C)	PRE (mm)	RAD (W m^−2^)	VPD (hPa)	TS (°C)	SWC (%)
TCZ	12.13 ± 3.9	1.91 ± 0.97	167.40 ± 25.06	4.96 ± 1.63	12.45 ± 4.54	22.69 ± 6.55
CCZ	4.96 ± 4.18	1.56 ± 0.78	142.26 ± 24.80	3.49 ± 1.06	6.57 ± 3.15	21.79 ± 8.0

Note: TCZ: temperate climate zone; CCZ: cold climate zone; TA: air temperature; PRE: precipitation; RAD: solar shortwave radiation; VPD: vapor pressure deficit; TS: soil temperature; SWC: soil water content. For each climate zone, the mean and standard deviation of each variable were calculated by first averaging daily observations within each site, then averaging across sites within the zone.

**Table 3:** Forest composition of the four geographical regions in the temperate (TCZ) and cold (CCZ) climate zones.

Region	Climate Zone	Sites	Dominant Forest Type	Local Tree Species Richness (Species ha^−1^)
East Asia	TCZ	4	Evergreen broadleaf; mixed broadleaf and conifer; plantations of Chinese fir and Masson pine	28.8 (26.8–30.7)
Europe	TCZ	16	Deciduous broadleaf; managed conifer stands	4.3 (3.7–4.6)
North America	TCZ	6	Temperate conifer; mixed conifer and broadleaf	3.0 (2.6–4.1)
Australia	TCZ	4	Temperate eucalypt forest and woodland	30.6 (26.3–31.6)
East Asia	CCZ	4	Deciduous conifer; mixed boreal and temperate	8.4 (6.7–9.9)
Europe	CCZ	11	Boreal and hemiboreal conifer	3.9 (3.6–4.1)
North America	CCZ	25	Boreal conifer	3.1 (2.8–5.8)

Note: TCZ: temperate climate zone; CCZ: cold climate zone. Sites is the number of flux towers retained in each region and zone (30 in the TCZ and 40 in the CCZ, 70 in total). Richness is summarized as the median, with the interquartile range in parentheses.

## Data Availability

All analyses in this study are based on openly available eddy covariance observations; the authors did not conduct additional field measurements. Daily meteorological drivers and GPP were derived from FLUXNET (https://fluxnet.org/, accessed on 1 November 2026), ChinaFLUX (http://www.chinaflux.org/, accessed on 1 November 2026) and the National Ecosystem Science Data Center of National Science and Technology Infrastructure of China (https://www.nesdc.org.cn/, accessed on 1 November 2026).

## Supplementary Materials

The following supporting information can be downloaded at: https://www.mdpi.com/article/10.3390/plants15213428/s1. Figure S1: Global feature importance of the environmental factors affecting GPP in East Asia, Europe, North America and Australia; Figure S2: The relative importance of the environmental factors on GPP based on the mean absolute SHAP values at each site; Figure S3: Significant pathways of environmental drivers on forest GPP in TCZ and CCZ; Figure S4: Pearson correlation matrix of driving variables with GPP in the temperate and cold climate zones. Table S1: Information of the eddy covariance sites used in this study; Table S2: Hyperparameter search spaces and optimal values for multiple machine learning models in simulating GPP.
