# Supplementary material for: Distinct Thresholds and Interactive Effects of Environmental Drivers on Forest Gross Primary Productivity Across Temperate and Cold Climate Zones
Source: Plants (Basel). 2026 Nov 4;15(21):3428. doi: 10.3390/plants15213428 (PMC13765888; doi:10.3390/plants15213428)
Supplement: Supplementary file 1 [file plants-15-03428-s001.zip › plants-4644323-supplementary.pdf]

Supplementary Materials

Distinct Thresholds and Interactive Effects of Environmental Drivers on Forest Gross Primary Productivity Across Temperate and Cold Climate Zones

Supplementary Figures

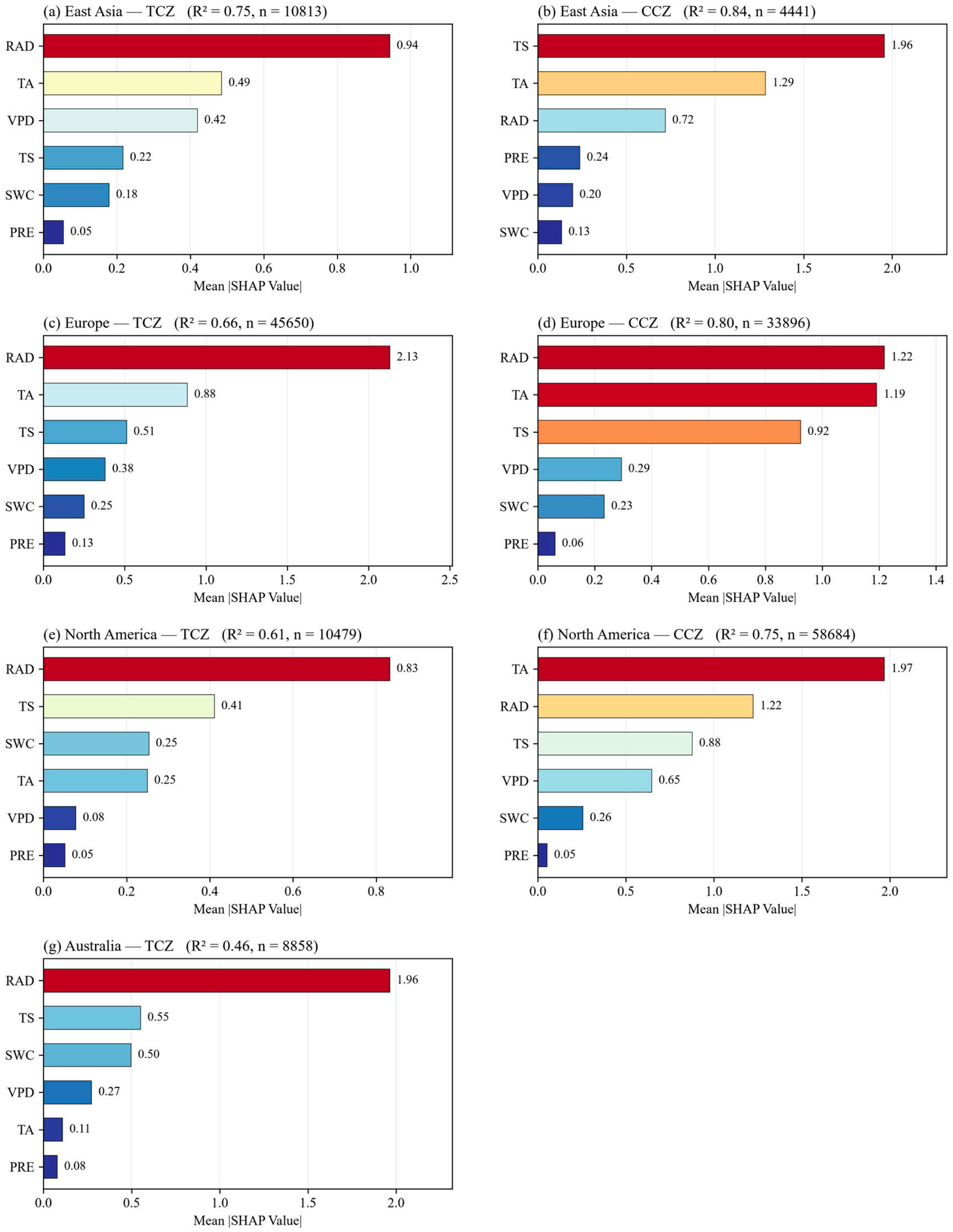

Figure S1. Global feature importance of the environmental factors affecting GPP in East Asia (a, b), Europe (c, d), North America (e, f) and Australia (g)

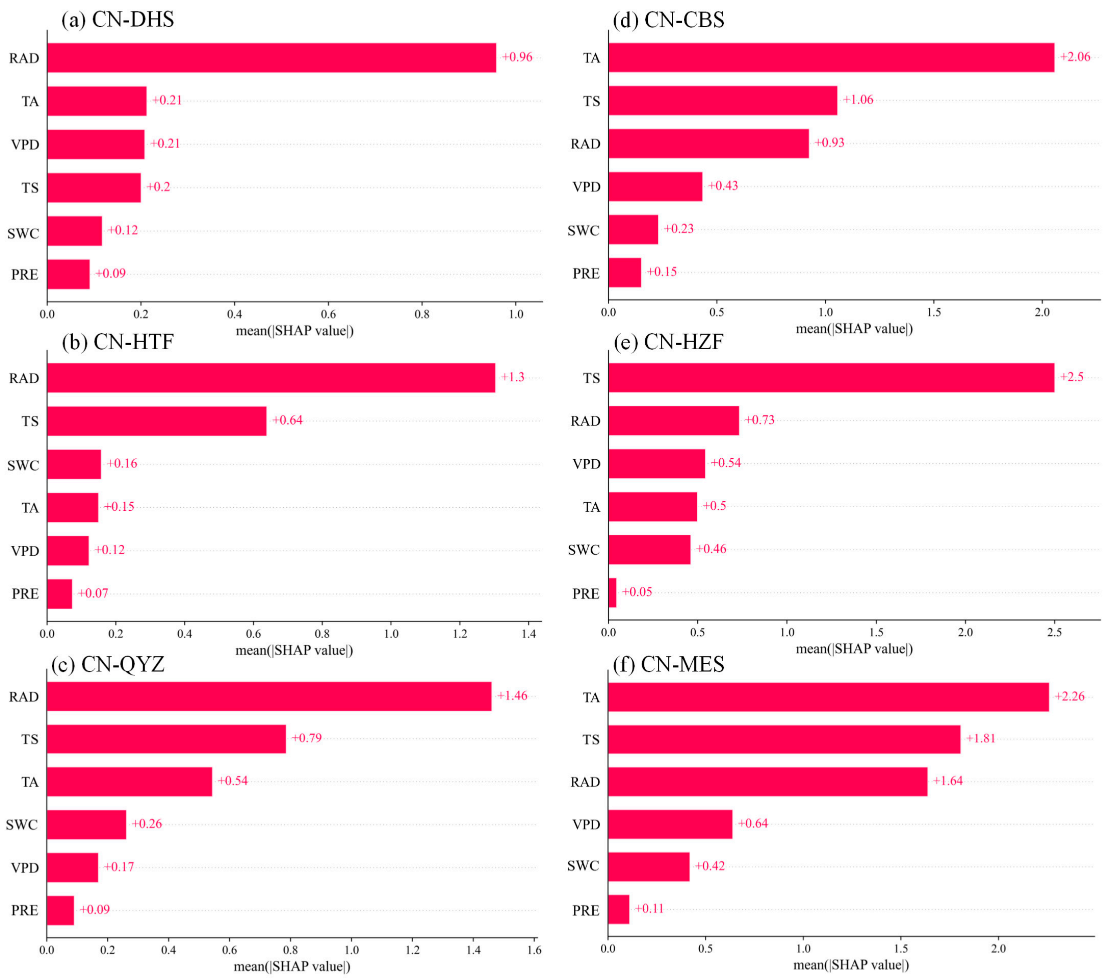

Figure S2. The relative importance of the environmental factors on GPP based on the mean absolute SHAP values at each site. CN-DHS, CN-HTF, CN-QYZ: forest sites in temperate climate zone; CN-CBS, CN-HZF, CN-MES: forest sites in cold climate zone

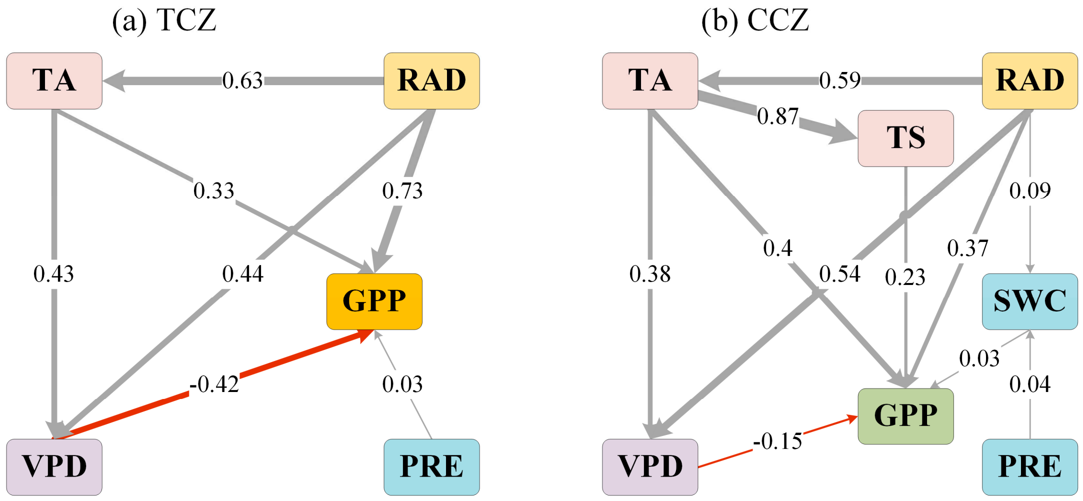

Figure S3. Significant pathways of environmental drivers on forest GPP in TCZ (a) and CCZ (b). Red and grey arrows indicate negative and positive paths, respectively. Arrow width is proportional to the standardized path coefficient. All path coefficients are statistically significant at $P < 0.05$. Fit metrics in TCZ: CFI = 0.93, TLI = 0.83, SRMR = 0.13, R^2 (GPP) = 0.49. CCZ: CFI = 0.96, TLI = 0.89, SRMR = 0.06, R^2 (GPP) = 0.57

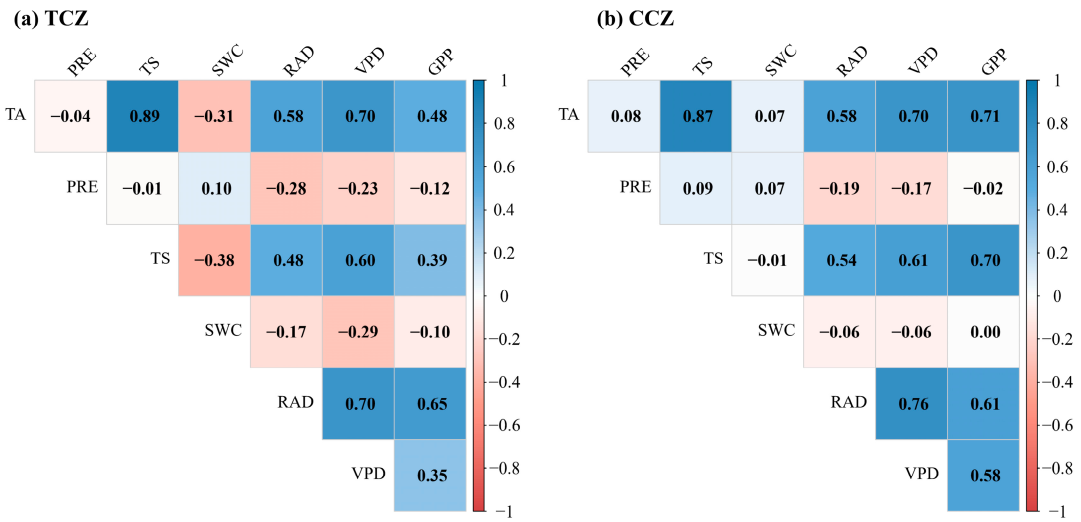

Figure S4. Pearson correlation matrix of driving variables with GPP in the temperate climate zone (TCZ) and the cold climate zone (CCZ)

Supplementary Tables

Table S1. Information of the eddy covariance sites used in this study

SITE_ID	Latitude	Longitude	TA (°C)	RAD (MJ m^{-2} d^{-1})	VPD (hPa)	PRE (mm)	TS (°C)	SWC (%)	Period	IGBP	Climate Zone	Tree species richness (species ha^{-1})
AU-Tum	-35.66	148.15	9.52	16.88	4.52	1.76	9.60	24.92	2001-2014	ENF	TCZ	31.4
AU-Wac	-37.43	145.19	10.70	12.27	4.04	5.73	10.97	30.17	2005-2008	EBF	TCZ	32.2
AU-Whr	-36.67	145.03	15.68	17.32	8.19	0.67	16.81	17.57	2011-2014	EBF	TCZ	15.7
AU-Wom	-37.42	144.09	11.38	15.58	3.85	2.01	11.97	20.82	2010-2014	EBF	TCZ	29.9
BE-Vie	50.30	6.00	8.25	9.92	2.62	2.12	8.17	30.37	1996-2014	MF	TCZ	4.4
CH-Lae	47.48	8.36	7.76	12.00	3.20	2.75	9.92	21.50	2004-2014	MF	TCZ	5.2
CN-DHS	23.17	112.54	19.90	11.85	5.98	3.47	20.12	21.54	2003-2010	EBF	TCZ	27.1
CN-HTF	26.79	109.59	15.23	9.97	5.59	3.10	16.81	25.39	2008-2012	ENF	TCZ	26.1
CN-QYZ	26.75	115.07	17.56	11.86	6.65	3.25	17.53	17.45	2003-2010	ENF	TCZ	30.5

SITE_ID	Latitude	Longitude	TA (°C)	RAD (MJ m^{-2} d^{-1})	VPD (hPa)	PRE (mm)	TS (°C)	SWC (%)	Period	IGBP	Climate Zone	Tree species richness (species ha^{-1})
DK-Sor	55.49	11.64	8.34	9.86	2.30	1.82	8.14	24.54	1996-2014	DBF	TCZ	n.a.
FR-LBr	44.72	-0.77	13.37	12.95	4.50	2.00	12.75	38.44	1996-2008	ENF	TCZ	4.0
IT-CA1	42.38	12.03	14.99	15.98	6.50	1.60	16.55	40.25	2011-2014	DBF	TCZ	n.a.
IT-CA3	42.38	12.02	14.76	15.92	6.43	1.53	17.31	19.29	2011-2014	DBF	TCZ	n.a.
IT-Col	41.85	13.59	7.24	14.84	3.30	2.51	5.74	31.54	1996-2014	DBF	TCZ	4.6
IT-Cp2	41.70	12.36	16.05	14.78	4.24	1.92	15.90	20.68	2012-2014	EBF	TCZ	3.6
IT-Cpz	41.71	12.38	15.12	15.37	5.49	1.51	14.64	16.54	1997-2009	EBF	TCZ	4.3
IT-Lav	45.96	11.28	7.02	13.68	2.77	2.58	5.66	36.69	2003-2014	ENF	TCZ	4.6
IT-PT1	45.20	9.06	14.04	13.52	4.40	1.41	13.02	28.59	2002-2004	DBF	TCZ	5.6
IT-Ro1	42.41	11.93	15.55	14.77	5.83	1.56	14.52	31.67	2000-2008	DBF	TCZ	3.7
IT-Ro2	42.39	11.92	15.47	15.44	6.21	1.49	12.82	28.18	2002-2012	DBF	TCZ	3.8

SITE_ID	Latitude	Longitude	TA (°C)	RAD (MJ m^{-2} d^{-1})	VPD (hPa)	PRE (mm)	TS (°C)	SWC (%)	Period	IGBP	Climate Zone	Tree species richness (species ha^{-1})
IT-SR2	43.73	10.29	15.47	14.48	3.86	2.58	15.57	16.56	2013-2014	ENF	TCZ	3.6
IT-SRo	43.73	10.28	15.22	15.03	4.20	1.66	14.41	17.93	1999-2012	ENF	TCZ	3.6
JP-SMF	35.26	137.08	14.71	13.52	6.13	2.77	14.22	7.12	2002-2006	MF	TCZ	31.2
NL-Loo	52.17	5.74	9.99	9.95	2.57	1.86	11.01	8.97	1996-2014	ENF	TCZ	4.8
US-Blo	38.90	-120.63	11.11	18.23	8.48	2.54	10.51	22.20	1997-2007	ENF	TCZ	6.7
US-KS1	28.46	-80.67	23.29	17.58	6.77	1.98	28.56	14.31	2002-2002	ENF	TCZ	n.a.
US-Me1	44.58	-121.50	9.13	14.90	6.28	0.37	12.05	14.61	2004-2005	ENF	TCZ	n.a.
US-Me2	44.45	-121.56	7.47	15.91	5.62	0.88	7.73	21.78	2002-2014	ENF	TCZ	3.2
US-Me4	44.50	-121.62	8.33	15.19	5.74	1.17	9.06	14.53	1996-2000	ENF	TCZ	2.7
US-Me5	44.44	-121.57	7.68	14.66	5.79	0.82	8.61	12.86	2000-2002	ENF	TCZ	2.3
CA-Gro	48.22	-82.16	3.23	11.83	3.50	1.73	5.48	40.81	2003-2014	MF	CCZ	4.3

SITE_ID	Latitude	Longitude	TA (°C)	RAD (MJ m^{-2} d^{-1})	VPD (hPa)	PRE (mm)	TS (°C)	SWC (%)	Period	IGBP	Climate Zone	Tree species richness (species ha^{-1})
CA-NS1	55.88	-98.48	0.93	10.81	3.88	0.55	0.97	28.23	2001-2005	ENF	CCZ	3.0
CA-NS2	55.91	-98.52	-2.75	10.14	2.98	0.65	0.79	14.02	2001-2005	ENF	CCZ	2.9
CA-NS3	55.91	-98.38	-2.15	10.67	2.92	0.35	2.78	12.60	2001-2005	ENF	CCZ	2.8
CA-NS4	55.91	-98.38	-1.41	10.77	3.37	0.49	1.37	21.56	2002-2005	ENF	CCZ	2.8
CA-NS5	55.86	-98.49	-1.81	10.73	2.93	0.53	2.43	12.69	2001-2005	ENF	CCZ	2.9
CA-Oas	53.63	-106.20	1.80	11.81	3.51	0.89	4.54	20.90	1996-2010	DBF	CCZ	3.1
CA-Qfo	49.69	-74.34	0.99	11.14	2.91	2.06	5.41	14.69	2003-2010	ENF	CCZ	2.2
CA-SF1	54.49	-105.82	-0.06	11.36	2.47	0.78	2.37	22.98	2003-2006	ENF	CCZ	3.0
CA-SF2	54.25	-105.88	0.20	11.86	3.03	0.48	2.70	13.00	2001-2005	ENF	CCZ	2.7
CA-TP1	42.66	-80.56	8.77	14.72	3.50	2.33	10.90	11.36	2002-2014	ENF	CCZ	6.2
CA-TP2	42.77	-80.46	8.60	13.96	3.19	2.00	7.74	9.19	2002-2007	ENF	CCZ	6.1

SITE_ID	Latitude	Longitude	TA (°C)	RAD (MJ m^{-2} d^{-1})	VPD (hPa)	PRE (mm)	TS (°C)	SWC (%)	Period	IGBP	Climate Zone	Tree species richness (species ha^{-1})
CA-TP3	42.71	-80.35	8.90	14.47	3.55	2.33	9.58	10.33	2002-2014	ENF	CCZ	6.1
CA-TP4	42.71	-80.36	8.54	14.00	2.81	2.33	9.89	11.92	2002-2014	ENF	CCZ	5.8
CA-TPD	42.64	-80.56	9.67	14.22	4.11	1.81	9.33	15.41	2012-2014	DBF	CCZ	6.7
CN-CBS	42.40	128.46	3.47	13.81	4.19	1.27	5.44	21.26	2003-2010	MF	CCZ	8.1
CN-HZF	51.78	121.02	-4.90	12.48	1.86	1.32	-1.11	14.04	2014-2018	DNF	CCZ	2.7
CN-MES	45.42	127.67	2.34	13.29	3.87	2.18	5.13	17.21	2016-2018	DBF	CCZ	13.8
CZ-BK1	49.50	18.54	6.82	10.27	2.52	2.80	5.77	24.07	2004-2014	ENF	CCZ	3.7
DE-Hai	51.08	10.45	8.34	10.45	3.29	1.62	7.93	34.84	2000-2012	DBF	CCZ	4.7
DE-Lkb	49.10	13.30	4.56	10.67	2.49	2.28	8.54	35.34	2009-2013	ENF	CCZ	4.0
DE-Lnf	51.33	10.37	8.17	10.57	3.18	1.33	6.70	35.37	2002-2012	DBF	CCZ	4.2
DE-Obe	50.79	13.72	6.49	10.21	2.59	2.28	6.19	32.91	2008-2014	ENF	CCZ	3.7

SITE_ID	Latitude	Longitude	TA (°C)	RAD (MJ m^{-2} d^{-1})	VPD (hPa)	PRE (mm)	TS (°C)	SWC (%)	Period	IGBP	Climate Zone	Tree species richness (species ha^{-1})
DE-Tha	50.96	13.57	8.77	10.74	3.92	1.85	8.02	19.77	1996-2014	ENF	CCZ	4.0
FI-Hyy	61.85	24.29	4.37	8.54	2.67	1.26	5.43	26.54	1996-2014	ENF	CCZ	3.6
FI-Sod	67.36	26.64	0.72	7.88	2.04	1.16	3.49	8.20	2001-2014	ENF	CCZ	3.4
IT-Isp	45.81	8.63	13.37	13.04	6.44	3.73	11.67	30.79	2013-2014	DBF	CCZ	5.9
IT-Ren	46.59	11.43	4.55	13.05	2.78	1.86	5.84	35.55	1998-2013	ENF	CCZ	2.6
JP-MBF	44.39	142.32	3.58	11.34	1.60	2.06	6.17	34.48	2003-2005	DBF	CCZ	8.7
RU-Fyo	56.46	32.92	5.17	9.40	2.80	1.18	5.60	35.66	1998-2014	ENF	CCZ	3.9
US-GLE	41.37	-106.24	0.02	16.24	3.69	3.18	3.42	25.09	2004-2014	ENF	CCZ	2.2
US-Me3	44.32	-121.61	8.13	15.88	6.06	0.73	10.52	12.10	2004-2009	ENF	CCZ	2.1
US-Me6	44.32	-121.61	7.69	15.86	5.85	0.78	8.88	11.29	2010-2014	ENF	CCZ	2.1
US-MMS	39.32	-86.41	12.37	14.44	5.34	2.02	11.82	36.57	1999-2014	DBF	CCZ	10.4

SITE_ID	Latitude	Longitude	TA (°C)	RAD (MJ m^{-2} d^{-1})	VPD (hPa)	PRE (mm)	TS (°C)	SWC (%)	Period	IGBP	Climate Zone	Tree species richness (species ha^{-1})
US-NR1	40.03	-105.55	2.29	16.20	4.45	1.49	2.68	11.64	1998-2014	ENF	CCZ	2.8
US-Oho	41.55	-83.84	10.51	13.94	4.69	1.47	11.83	18.65	2004-2013	DBF	CCZ	8.2
US-Syv	46.24	-89.35	4.41	13.83	3.51	1.31	7.44	23.46	2001-2014	MF	CCZ	5.4
US-UMB	45.56	-84.71	7.16	13.29	3.99	1.11	8.44	10.90	2000-2014	DBF	CCZ	5.7
US-UMd	45.56	-84.70	7.14	13.42	3.77	1.57	8.27	10.87	2007-2014	DBF	CCZ	5.1
US-WCr	45.81	-90.08	5.53	13.04	2.62	1.29	7.81	32.58	1999-2014	DBF	CCZ	5.7

Note: Data from six of the flux towers (CN-DHS, CN-HTF, CN-QYZ, CN-CBS, CN-HZF, and CN-MES) originate from the ChinaFLUX and National Ecosystem Science Data Center, while data from the remaining towers are from FLUXNET. Tree species richness is the modelled mean number of tree species per hectare in the 0.025° (about 2.5 km) grid cell containing each tower, taken from the global forest tree species richness map of Liang et al. (2022) [76].

Table S2. Hyperparameter search spaces and optimal values for multiple machine learning models in simulating GPP

Model	Key parameter	Search Space	Optimal value for TCZ	Optimal value for CCZ
XGBoost	learning_rate	[0.001, 0.005, 0.01, 0.05, 0.1, 0.15, 0.2]	0.05	0.01
	n_estimators	[50, 100, 200, 300, 500, 800, 1000]	100	500
	max_depth	[2, 3, 4, 5, 6, 7, 8, 9, 10]	9	4
	min_child_weight	[1, 2, 3, 4, 5, 7, 10]	2	1
	subsample	[0.6, 0.65, 0.7, 0.75, 0.8, 0.85, 0.9, 0.95, 1.0]	0.95	1
	reg_lambda	[0.005, 0.01, 0.05, 0.1, 0.2, 0.5, 1, 5, 10, 50]	5	0.01
	reg_alpha	[0.01, 0.1, 0.5, 1, 2, 5, 8, 10, 50]	5	10
	colsample_bytree	[0.6, 0.65, 0.7, 0.75, 0.8, 0.85, 0.9, 0.95, 1.0]	1	0.85
RF	n_estimators	[50, 100, 200, 300, 400, 500]	50	300
	max_depth	[1, 2, 5, 10, 20, 30, 40]	20	5
	max_features	['auto', 'sqrt', 'log2']	log2	log2
	min_samples_split	[2, 5, 10, 15, 20]	10	2
	min_samples_leaf	[1, 2, 3, 4, 5]	4	2
	bootstrap	[True, False]	True	True
GBDT	learning_rate	[0.001, 0.005, 0.01, 0.05, 0.1, 0.2, 0.3]	0.1	0.01
	n_estimators	[50, 100, 200, 300, 500, 800, 1000]	300	300
	max_depth	[2, 3, 5, 7, 10, 15, 20]	3	5
	min_samples_split	[2, 5, 10, 15, 20]	10	10
	min_samples_leaf	[1, 2, 3, 4, 5]	2	2
	subsample	[0.5, 0.6, 0.7, 0.8, 0.9, 1.0]	1	0.8
	max_features	['sqrt', 'log2', None]	log2	sqrt
LightGBM	n_estimators	[50, 100, 200, 300, 500, 800, 1000]	50	100
	learning_rate	[0.001, 0.01, 0.05, 0.1, 0.2, 0.3]	0.2	0.05
	num_leaves	[31, 50, 100, 150, 200]	50	100
	reg_lambda	[0, 0.1, 0.5, 1, 5]	1	1
	reg_alpha	[0, 0.1, 0.5, 1, 5]	5	0
	subsample	[0.5, 0.6, 0.7, 0.8, 0.9, 1.0]	0.6	0.9
SVR	kernel	['rbf', 'linear']	rbf	rbf

Model	Key parameter	Search Space	Optimal value for TCZ	Optimal value for CCZ
	C	[0.01, 0.1, 1, 10, 100]	0.1	10
	gamma	[0.001, 0.01, 0.1, 1, 'scale']	0.1	0.01
	epsilon	[0.01, 0.05, 0.1, 0.2, 0.5]	0.5	0.1
ANN	hidden_layer_sizes	[(50,), (100,), (50, 50), (100, 50), (100, 100)]	(50,)	(100, 50)
	activation	['relu', 'tanh']	relu	relu
	learning_rate_init	[0.001, 0.01, 0.1, 0.2]	0.1	0.1
	alpha	[0.001, 0.01, 0.1, 0.2, 0.5]	0.001	0.1
	batch_size	[8, 16, 32, 64, 128, 256]	128	32
	max_iter	[300, 500, 800, 1000, 1500]	1000	500
